# Diversity and Pathogenicity of *Neopestalotiopsis* Species Associated with Strawberry Leaf Spot and Fruit Rot in Nova Scotia

**DOI:** 10.3390/jof12040275

**Published:** 2026-04-10

**Authors:** Sajid Rehman, Shawkat Ali

**Affiliations:** 1 Western Crop Innovations, Lacombe, AB T4L 1W8, Canada; srehman@westerncropinnovations.com; 2 Perennia Food and Agriculture Inc., Kentville, NS B4N 1J5, Canada; 3 Kentville Research and Development Centre, Agriculture and Agri-Food Canada, Kentville, NS B4N 1J5, Canada

**Keywords:** strawberry, leaf spot, fruit rot, *Neopestalotiopsis*

## Abstract

We reported the first isolation and characterization of *Neopestalotiopsis* spp. from symptomatic strawberry plants in Nova Scotia, Canada. Morphological and multilocus sequence analyses confirmed that these isolates were closely related to previously identified aggressive *Neopestalotiopsis* spp. strains from strawberry and blueberry in the southeastern United States and other countries. Five representative isolates were evaluated for pathogenicity on detached leaves, whole plants, and fruits of multiple strawberry cultivars. The results revealed significant variation in virulence, with isolate NS-1 causing the most severe necrosis across all tissue types. Statistical analysis revealed significant effects of isolate, cultivar, and their interaction on disease severity, indicating differential cultivar responses to the tested isolates. Notably, tissue-specific differences were observed, with some isolates being aggressive on leaves but less virulent on fruit or whole plants, reinforcing the importance of multi-organ phenotyping in resistance screening. Phylogenetic analysis clustered the Nova Scotia isolates within the same clade as *Neopestalotiopsis* isolate 17–43 L from strawberry and isolates from blueberry, suggesting a potential epidemiological link. The shared nursery propagation system of strawberries and blueberries raises the risk of cross-infection, posing a substantial challenge to disease management strategies in both crops. Collectively, these findings underscore the urgent need for continued surveillance, population-level pathogen analysis, and the development of resistant cultivars to mitigate the spread of this emerging and rapidly evolving pathogen.

## 1. Introduction

Strawberry (*Fragaria x ananassa* Duch.) is an important horticultural crop in Canada. It was cultivated on 10,129 acres in 2021, with total production of 25,893 tonnes, and generated a farm gate value of CAD 128.5 million. Nova Scotia ranks third in strawberry production nationally following Ontario and Quebec, with a farm gate value of CAD 13.2 million in 2022 [1]. In addition, Nova Scotia’s strawberry nursery is valued at approximately CAD 9 million, and supplies plants not only for fruit production in Canada but also to growers in the southern and northeastern United States.

As a member of the Rosaceae family, strawberry can be infected by different fungal, bacterial, and viral pathogens affecting plants at both the nursery and fruit-production stages. In Nova Scotia, several fungal pathogens are of particular concern in strawberry production, including powdery mildew (*Podosphaera aphanis*), anthracnose fruit rot (*Colletotrichum acutatum*), leaf spot (*Mycosphaerella fragariae*), verticillium wilt (*Verticillium dahliae*), crown rot (*Phytophthora cactorum*), gray mold (*Botrytis cinerea*), and black root rot (caused by a complex of *Pythium*, *Rhizoctonia*, and *Fusarium* spp.). Several species of genus *Pestalotiopsis* have been reported as causal agents of leaf blights, shoot dieback, and fruit rot on a wide range host plants [2,3]. Recently, based on morphological characteristics and molecular sequence analysis, the genus *Pestalotiopsis* has been divided into three distinct genera: *Neopestalotiopsis, Pseudopestalotiopsis,* and *Pestalotiopsis* [2]. *Neopestalotiopsis rosae* (formerly *Pestalotiopsis longisetula* and, prior to that, *Pestalotia longisetula*) caused significant losses in commercial strawberry fields in 1972 in Florida [4]. Since then, this pathogen has been reported to cause root and crown rot in strawberry in many countries, including Argentina, Bangladesh, Belgium, Egypt, Mexico, and Spain [5,6,7,8,9,10]. Its characteristic above-ground symptoms includes stunting, wilting, and necrosis of leaves. In addition, the below-ground symptoms include darkening of roots and brownish discoloration inside the crown, and eventually the entire plant collapses and dies [6,8]. In 2021, Baggio et al. [11] reported an outbreak of leaf spot and fruit rot in Florida strawberries, which was caused by *Neopestalotiopsis* spp., which is phylogenetically similar to *N. rosae.* The first outbreak was reported in 2017 in Florida, where entire strawberry fields were destroyed due to severe blighted lesions on leaves and fruits. By 2020, the disease had spread further, affecting 80 hectares of strawberry fields across 18 growers. Unlike *N. rosae*, *Neopestalotiopsis* spp. primarily cause symptoms on above-ground tissues, particularly leaves and fruits. Furthermore, the leaf spots were accompanied by rapid necrosis of entire leaves, on which black acervuli could be observed. The pathogen’s spores are easily dispersed by rain droplets, and disease epidemics are favored by warm and humid weather conditions [12,13].

During the 2021 cropping season, several strawberry plants showing necrotic leaves were collected from fields in the Annapolis Valley and Truro regions in Nova Scotia. The collected plants appeared stunted, with a mix of asymptomatic and necrotic leaves, and tested negative for all major known strawberry diseases. The main objective of this study was to identify the causal organism associated with these symptoms and to investigate the molecular and pathogenic diversity of the pathogen present in the infected strawberry samples.

## 2. Materials and Methods

### 2.1. Fungal Isolates

During the cropping season of 2021, approximately 31 strawberry samples were submitted to the plant health lab at Perennia by six major strawberry growers from the Annapolis Valley and Truro. The submitted strawberry plants exhibited stunted growth, and a mix of asymptomatic and necrotic leaves. Initial diagnostic testing returned negative results for all major known strawberry pathogens. To identify the causal organism, infected leaves were cut into 0.5–1.0 cm pieces, followed by surface sterilization in 70% ethanol for 30 s and 5% sodium hypochlorite for 1 min, and rinsed 3 times with sterile distilled water. The sterilized tissues were then dried on two layers of sterile Whatman filter paper. These leaf segments were incubated on 1.5% potato dextrose agar (PDA) plates supplemented with Streptomycin (50 µg/mL), as well as on moist sterile filter paper placed in a transparent plastic container under a 12 h dark/12 h light photoperiod at 22 ± 2 °C. After 7 to 10 days post inoculation (dpi), the fungal growth was observed, and 19 single-conidial isolates were obtained and designated NS-1 to NS-19. Five representative isolates (NS-1 to NS-5) were selected for detailed molecular and pathogenic characterization. All isolates will be submitted to the Canadian Collection of Fungal Cultures (DAOMC).

### 2.2. Morphological Identification

For microscopic characterization, conidia were mounted in sterile distilled water and examined using an Olympus CXKX41 compound microscope (Olympus Corporation, Tokyo, Japan) at 400× and 1000× magnifications. Digital images were captured and morphometric measurements (conidial length, width, septation, and appendage length) were obtained using cellSens imaging software (version 3.1, Olympus Corporation). Measurements were taken from at least 100 conidia per isolate (*n* = 100), and size ranges were recorded. Morphological characteristics were compared with published descriptions of *Neopestalotiopsis* species [12,14]. Acervuli formation on infected leaf tissues and PDA cultures was examined using an Olympus SZX12 stereomicroscope (Olympus Corporation, Japan). Digital images were captured, and acervuli dimensions were measured using cellSens imaging software (version 3.1, Olympus Corporation). Measurements were obtained from at least 30 acervuli per isolate (*n* = 30), and size ranges were recorded.

### 2.3. Molecular Characterization

Five single conidial isolates were sub-cultured on PDA plates and incubated for seven days under a 12 h light/12 h dark photoperiod of at 22 ± 2 °C. A mixture of mycelium and black acervuli was harvested using a sterile loop, and the genomic DNA was extracted from five isolates using a Plant/Fungal DNA Isolation Kit (Norgen Biotek Corp., Thorold, ON, Canada) according to the manufacturer’s instructions.

The partial sequences of the internal transcribed spacer (*ITS*) region of ribosomal DNA, the translation elongation factor 1-α (*TEF1-α*) region, and the *β-tubulin* region (*β-tub*) were amplified by polymerase chain reaction (PCR) using ITS1/ITSF primers [15], EF1-up/EF1-low primers [16], and Bt2a/Bt2b primers [17], respectively. Each 25 µL PCR reaction mixture contained 12.5 µL of 2X FroggaBio PCR Master Mix (containing Taq DNA Polymerase, dNTPs, Mg^2+^, Reaction Buffer, and an inert loading dye; FroggaBio Scientific solutions), 3 µL of template DNA, and 7.5 µL PCR water. The thermocycler was programmed with initial denaturation at 94 °C for 3 min, with 35 subsequent cycles of 94 °C for 30 s, 58 °C for 30 s, and 72 °C for 1 min, and a final extension of 72 °C for 5 min. To confirm successful amplification and verify the size of the amplified DNA fragments, all amplicons were electrophoresed on 1% agarose gel using 1× Tris-borate-EDTA (TBE) running buffer. For sequencing, the PCR products were cleaned up using ExoSAP-IT PCR Product Cleanup Reagent (Applied Biosystems, Foster City, CA, USA) to remove dNTPs and primers. The cleaned PCR products were sequenced from both ends at Eurofins Genomics (Louisville, KY, USA) using BigDye 3 Terminator Cycle sequencing chemistry (Applied Biosystem’s 3730xl DNA Analyzer Technology). The sequences generated using forward and reverse primers were assembled and trimmed using SnapGene software 4.3.11 (https://www.snapgene.com/) for all target genes. Nucleotide sequences were deposited in GenBank (*ITS*, ON454613 to ON454617; *β-tub*, ON464179, and ON549866 to ON549869; and *TEF1-α*, ON631213 to ON631217). For phylogenetic analysis, concatenated sequences of the *ITS, β-tub*, and *TEF1-α* regions from five isolates (NS-1 to NS-5), were aligned with sequences of 31 *Pestalotiopsis* and *Neopestalotiopsis* spp. obtained from NCBI. The evolutionary history was inferred using the Maximum Likelihood method and Tamura–Nei model in MEGA X [18,19].

For molecular identification and in silico analysis, *β-tub* gene sequences from the Nova Scotia isolates (NS-1–NS-5) were compared with virulent strawberry isolate from Florida (17–43 L), three blueberry isolates from Georgia (CB22-023, CB22-027, and CB22-028), and *N. rosae* CBS-101057. The final aligned sequences were assembled in the MultAlin interface and screened for point mutation in the new variant strains compared to *N. Rosae*.

### 2.4. Pathogenicity Tests

The pathogenicity assays were conducted on detached leaves, strawberry fruits, and whole plants as described by Karimi et al. [20]. Detached-leaf assay: The pathogenicity of four *Neopestalotiopsis* spp. isolates (NS-1, NS-2, NS-3, and NS-4) was tested on detached leaves. All pure isolates were maintained on PDA, and 12–14-day-old cultures were used to prepare the inoculum by rubbing the agar surface with a sterile glass slide, followed by filtration through two layers of sterile cheese cloth. The final conc. was adjusted to 10^4^ conidia per mL, and Tween 20 was added to a final conc. of 0.01% just before inoculation. Strawberry plants of two varieties, Seascape and Albion, were maintained in an AAFC growth chamber at 20 ± 2 °C with a photoperiod of 16 h light/8 h dark. For each test, 6 triplet leaves of strawberry in two replications were placed on sterile, moist filter paper in transparent plastic containers and inoculated with 5 mL spore suspension with a handheld sprayer, followed by incubation in a growth chamber at 22 ± 2 °C with a photoperiod of 12 h light/12 h dark and 70–80% relative humidity. Control leaves were sprayed with sterile distilled water containing 0.01% Tween 20 and incubated under identical conditions. No necrotic lesions developed on the control leaves. Leaf necrosis (%) representing the proportion of the leaf area showing necrotic lesions was assessed at 10 dpi using ImageJ software version 1.53 [21]. For each leaf, the total leaf area and the necrotic lesion area were quantified using color thresholding. Disease severity was calculated as the percentage of necrotic area relative to the total leaf area of the same leaf. This approach accounts for differences in leaf size among samples.

Plant inoculation: The pathogenicity of four isolates (NS-1–NS-4) was tested on the plants of the strawberry variety Honeoye. To test each isolate, bare-root strawberry plantlets were grown individually in 12 cm diameter pots containing a commercial peat-based potting mix (Pro-Mix BX, Premier Tech, Rivière-du-Loup, QC, Canada). Plants were maintained in Conviron growth chambers at 22 ± 2 °C with a photoperiod of 16 h light/8 h dark. Furthermore, the plants were irrigated daily during daylight hours except during the incubation period after inoculations. Plants of about 21 days old were inoculated with 15 mL spore suspension (10^4^ spores/mL) using a handheld sprayer, followed by incubation at 22 ± 2 °C with a photoperiod of 12 h light/12 h dark. A ~100% relative humidity was maintained in the growth chamber for 72 h. Afterwards, the inoculated plants were incubated at 22 ± 2 °C with a photoperiod of 16 h light/8 h dark and 70–80% relative humidity as described above. The control strawberry plants were sprayed with deionized water only. After 14 days of inoculation, strawberry plants were evaluated for disease incidence (number of symptomatic plants) and disease severity, expressed as the percentage of necrotic area on the first five leaves. These percentage disease severity values were used for statistical analysis. For descriptive purposes only, disease severity was also classified according to the James scale [22] into five categories: 1 = 1–10% (resistant—R), 2 = 11–25% (moderately resistant—MR), 3 = 26–50% (moderately susceptible—MS), 4 = 51–80% (susceptible—S), and 5 = 81–100% (highly susceptible—HS). Disease scores of 0–2 were broadly considered resistant, and 3–5 susceptible [23]. One-way ANOVA was performed using the percentage disease severity data to detect differences among isolates.

Fruit inoculation: Strawberry fruits at the commercial red-ripe stage (uniform red coloration over ≥90% of the fruit surface, firm texture, and absence of visible defects) were purchased from a local supermarket and used within 24 h of purchase. Their calyces were removed, followed by surface sterilization in 70% ethanol for 1 min; then, they were rinsed once in sterile water for 2 min and in 10% sodium hypochlorite for 2 min, and rinsed twice in sterile water for 2 min, followed by air-drying in a biological safety cabinet. For inoculation with each isolate, 10 non-wounded strawberry fruits were placed on sterile Petri dishes in a transparent plastic container on a layer of wet sterile filter papers, followed by point inoculation with 25 µL of spore suspension using a 10 µL micropipette, and incubated under the same conditions as described above. As a control, strawberries were mock inoculated with 25 µL of sterile water. Disease incidence (number of symptomatic fruits) and disease severity were recorded at 4, 7 and 10 days post inoculation. Disease severity was estimated visually as the percentage of fruit surface area covered by necrotic lesions relative to the total fruit surface area, and each fruit was evaluated individually. The experiment was repeated twice.

All experiments were arranged in a completely randomized design. Detached-leaf assays were conducted with two biological replications, each consisting of six triplet leaves per treatment. Whole-plant and fruit inoculation experiments were conducted twice independently. For detached-leaf assays, percentage necrosis data were analyzed using a two-way analysis of variance (ANOVA) with cultivar and isolate as fixed effects. Whole-plant and fruit assays were analyzed using one-way ANOVA to evaluate differences among isolates. When experiments were repeated, data from independent runs were combined after confirming homogeneity of variance. Disease severity percentages were used for statistical analyses. Means were separated using Tukey’s Honestly Significant Difference (HSD) test at α = 0.05. Prior to ANOVA, normality and homogeneity of variance were evaluated using Shapiro–Wilk and Levene’s tests, respectively. All statistical analyses were performed using Python version 3.13 (statsmodels package version 0.14.0), and figures were generated using matplotlib version 3.8.0.

## 3. Results

### 3.1. Morphological Characterization

**Figure 1:**
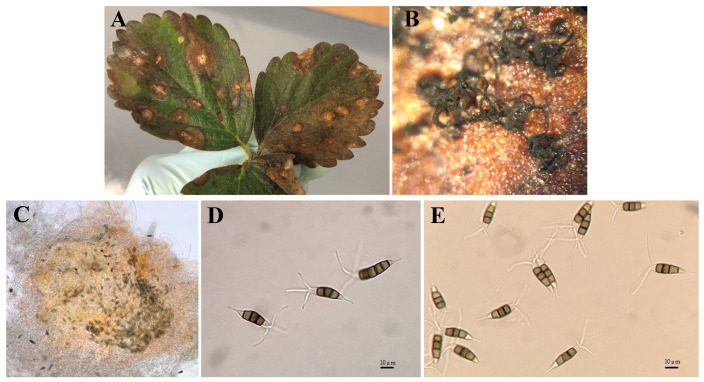
A sample of *Neopestalotiopsis* spp.-infected strawberry leaves with necrotic lesions bearing acervulli (**A**); erupted acervulli bearing cirri under 40× magnification (**B**); a conidiomata with conidia (**C**); conidia of NS-2 (**D**) and NS-1 (**E**) isolates of *Neopestalotiopsis* spp.

**Figure 2:**
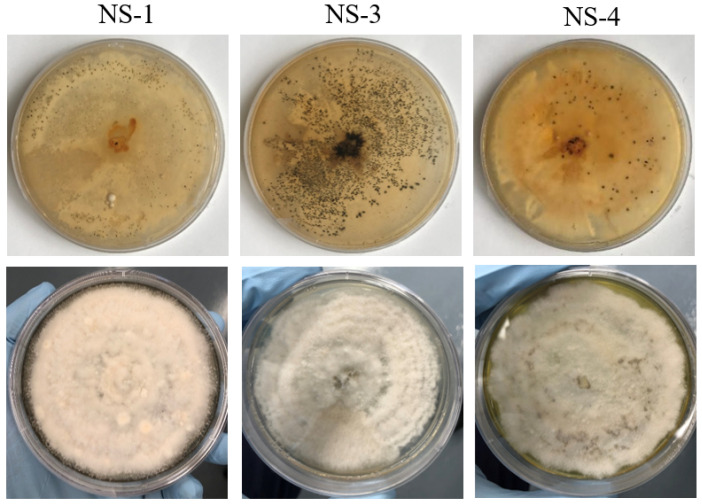
Growth of three isolates of *Neopestalotiopsis* spp. (NS-1, NS-3, and NS-4) on PDA. The lower panel displays fungal growth from the upper surface of the PDA plate, and the upper panel displays yellowish growth from the reverse side of the PDA plate.

After 7 to 10 days post incubation of infected leaves on PDA, black acervuli measured 280.3 ± 6.4 µm in diameter (270–290 µm), and black cirri were observed on the surface of leaf segments (Figure 1A–C). Microscopic observations revealed fusiform-to-ellipsoid conidia, measuring 24.3 ± 1.6 × 7.6 ± 0.5 µm (21.5–27.3 × 6.8–8.5 µm (n = 100)), with four septa (five cells). Apical appendages (2–4 per conidium) measured 20.4 ± 1.8 µm (18.5–23.5 µm) and one basal appendage measured 11.2 ± 2.4 µm (7.5–16.5 µm). Among the five cells, basal and apical cells were hyaline, with the second and third apical cells being dark brown and the fourth cell being light brown in color (Figure 1D,E). Based on the acervuli and conidia morphology, the causal agent was identified as *Neopestalotiopsis* spp. Nineteen single-spore isolates were prepared on PDA under aseptic conditions. Initially, fungal isolates grew on PDA in a cottony white form from the upper surface; however, with the formation of acervuli (7–14 days), the color of some of the isolates turned yellowish from pigmentation on the reverse side (Figure 2).

### 3.2. Molecular Characterization

**Figure 3:**
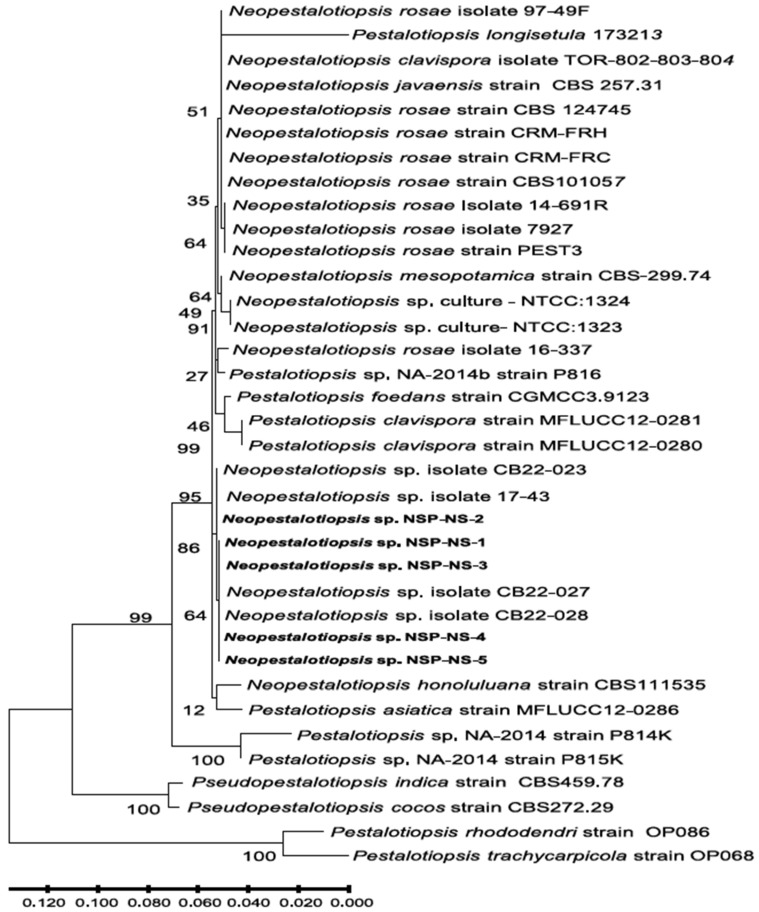
Phylogenetic relationships of five isolates of *Neopestalotiopsis* spp. (NS-1 to NS-5 in bold) with other *Pestalotiopsis* and *Neopestalotiopsis* spp. based on concatenated sequences of the internal transcribed spacer 2 (*ITS*) region, *β-tub*, and *TEF1-α* sequences. The sequences of 31 *Pestalotiopsis* and *Neopestalotiopsis* spp. were obtained from NCBI. The evolutionary history was inferred by using the Maximum Likelihood method and Tamura–Nei model [18]. The tree with the highest log likelihood (−3117.29) is shown. The initial tree(s) for the heuristic search were obtained automatically by applying the Neighbor-Join and BioNJ algorithms to a matrix of pairwise distances estimated using the Maximum Composite Likelihood (MCL) approach and then selecting the topology with the superior log likelihood value. The tree is drawn to scale, with branch lengths measured as the number of substitutions per site. This analysis involved 36 nucleotide sequences. The codon positions included were 1st + 2nd + 3rd + Noncoding. There were a total of 811 positions in the final dataset. Evolutionary analyses were conducted in MEGA X [18].

To confirm the identity at the DNA level, three genomic regions (the internal transcribed spacer (*ITS*) region of ribosomal DNA (rDNA), translation elongation factor 1-α (*TEF1-α*) region, and (*β-tub*) region) were amplified by PCR and sequenced from five *Neopestalotiopsis* spp. isolates. Sequences of these isolates for *ITS*, *TEF1-α*, and *β-tub* were submitted to GenBank with accession numbers for *ITS* (ON454613 to ON454617), *TEF1-α* (ON631213 to ON631217), and *β-tub* (ON464179 and ON549866 to ON549869). The partial sequences of these three gene regions of the four isolates from Nova Scotia were identical, while one isolate, NS-2, had 10 bp variation in the TEF sequence from the other four isolates (Figure S1). Interestingly, several of these mutations are similar to those found in *N. roase* and one of the *Neopestalotiopsis* spp. isolates from blueberry in Georgia [24]. BLASTn (Nucleotide BLAST: Search nucleotide databases using a nucleotide query, https://blast.ncbi.nlm.nih.gov/Blast.cgi?PROGRAM=blastn&PAGE_TYPE=BlastSearch&LINK_LOC=blasthome) analysis (accessed on 11 November 2025) of the *ITS* region and beta-tubulin gene showed 100% identity to *Neopestalotiopsis* spp. isolate 17–43 L, isolated from strawberry (accession nos. MK895144 and MK903340 respectively), and to the recent isolates of *Neopestalotiopsis* spp. from Blueberry in Georgia [24]. Furthermore, the phylogenetic analysis based on concatenated sequences of the *ITS* region, *β-tub*, and *TEF1-α* confirm the BLAST results, and showed that the Nova Scotia isolates clustered in the same clade as *Neopestalotiopsis* spp. isolate 17–43 L from strawberry, and the blueberry isolate of *Neopestalotiopsis* spp. (Figure 3).

The *β-tub* gene sequences from the Nova Scotia isolates (NS-1 to NS-5) were identical to those of the virulent strawberry isolate from Florida (17–43 L) and three blueberry isolates from Georgia (CB22-023, CB22-027, CB22-028) (Figure S2). All these isolates have point mutations that create restriction enzyme cut sites (CTTCCGGTA) for restriction endonuclease enzyme BsaW1 [25]. This single point mutation at the target site 294 bp is unique to *Neopestalotiopsis* spp., which was found in *N. rosae* CBS-101057.

### 3.3. Strawberry Detached-Leaf Necrosis Analysis

**Figure 4:**
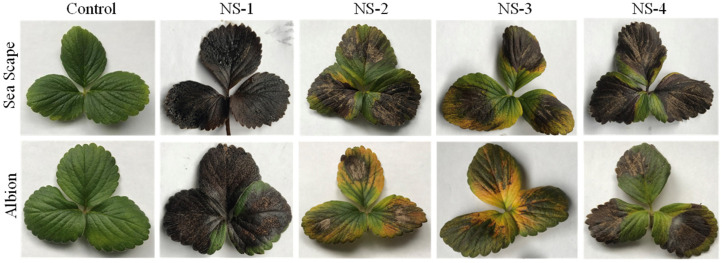
Detached strawberry leaves of the cultivars Seascape and Albion infected with four isolates of *Neopestalotiopsis* spp. (NS-1, NS-2, NS-3, and NS-4) at 10 days post inoculation. Detached leaves in the control were treated with sterile water only. Disease symptoms like dark brown necrotic lesions containing acervulli are evident on the infected leaves.

**Figure 5:**
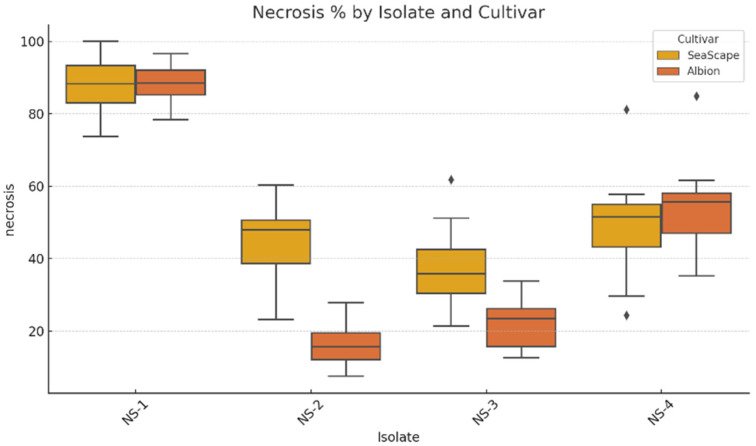
Boxplot showing the distribution of percentage necrotic leaf area in strawberry cultivars “Albion” and “Seascape” infected by four *Neopestalotiopsis* isolates (NS-1 to NS-4). Each box represents the interquartile range (IQR), with the horizontal line indicating the median. Whiskers represent 1.5× IQR, and individual rhombus-shaped points represent outliers.

All four isolates displayed diverse reactions on the detached leaves of the strawberry cultivars Seascape and Albion. On leaves, characteristic necrotic lesions bearing black acervuli of *Neopestalotiopsis* spp. with or without chlorosis (depending on the variety) were observed after 7 to 10 dpi (Figure 4). On Seascape, the average percentages of leaf necrosis were 87.7% (HS) for NS-1, 45.4% (MS) for NS-2, 37.6% (MS) for NS-3, and 49.3% (MS) for NS-4 (Figure 5). Likewise, the average percentages of leaf necrosis on Albion leaves were 88.1% (HS) for NS-1, 16.5% (MR) for NS-2, 21.7% (MR) for NS-3, and 54.6% (S) for NS-4. The NS-1 isolate was the most aggressive isolate, both on the leaves of Seascape and Albion (Figure 4 and Figure 5).

**Figure 6:**
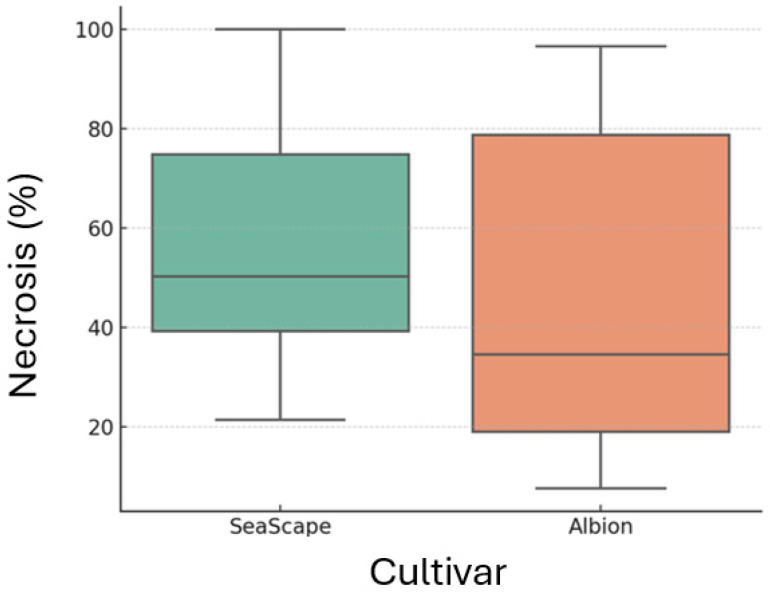
Boxplot comparing necrotic leaf area percentage between strawberry cultivars “Albion” and “Seascape”, averaged across all fungal isolates (NS-1 to NS-4). Differences in central tendency and spread reflect overall cultivar response to disease severity.

The two-way ANOVA revealed significant effects of isolate (F = 181.97, *p* < 0.0001), cultivar (F = 23.58, *p* < 0.00001), and their interaction (F = 15.07, *p* < 0.000001) on leaf necrosis (Table 1). The significant interaction between cultivar and isolate suggested the presence of isolate-specific resistance or susceptibility. Furthermore, Tukey’s HSD test showed significant differences among isolates, while the difference between ‘Albion’ and ‘Seascape’ was not statistically significant (*p* = 0.0741) (Figure 6).

### 3.4. Strawberry Leaf Necrosis Analysis on Potted Plants

**Figure 7:**
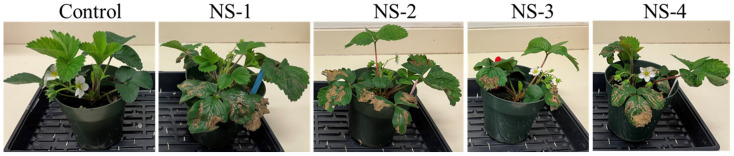
Strawberry leaves of the cultivar Honeoye infected with four isolates of *Neopestalotiopsis* spp. (NS-1, NS-2, NS-3, and NS-4). The strawberry plants in the control (on left side) were inoculated with sterile water. Infected plants display symptoms like light brown necrotic lesions containing acervulli on the infected leaves. Pictures of the infected and control plants were taken at 14 days post inoculation. The original images contain the control plant adjacent to each inoculated plant strain for visual comparison.

**Figure 8:**
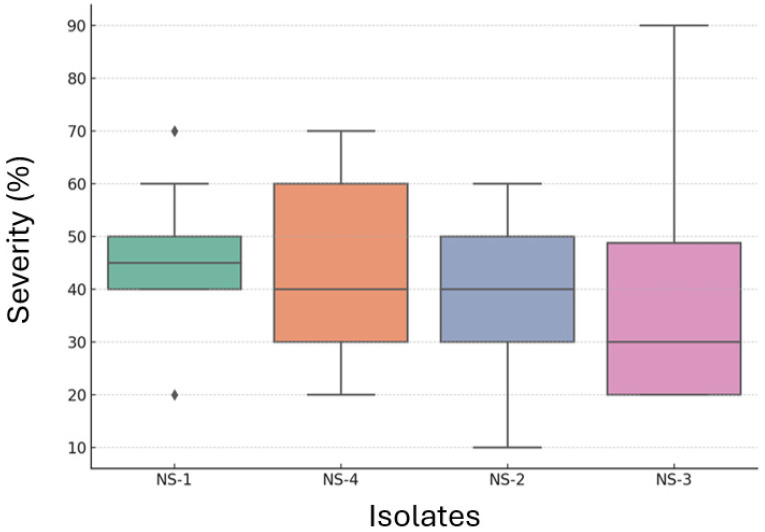
Boxplot showing average severity of necrotic leaf area in strawberry plants of cultivar “Honeoye” inoculated with different *Neopestalotiopsis* spp. isolates (NS-1 to NS-4). Each box represents the interquartile range (IQR), the horizontal line indicates the median, and whiskers represent 1.5× IQR. Individual rhombus-shaped points represent outliers.

Different *Neopestalotiopsis* spp. isolates differed in terms of pathogenicity on the leaves of strawberry plants (Figure 7). The *Neopestalotiopsis* spp. isolate NS-1 had the highest disease severity of 47.3% (MS), followed by NS-4 (43.9% (MS)), and NS-2 (37.2% (MS)). The isolate NS-3 had the lowest disease severity of 36% (MS) (Figure 8). In addition, the one-way ANOVA indicated a significant difference among isolates (F = 7.92, *p* = 5.24^−5^), suggesting that disease severity varied depending on the isolate (Table 2). Furthermore, Tukey’s HSD post hoc test revealed that several isolate pairs had statistically significant differences in mean disease severity.

### 3.5. Fruit Inoculation

**Figure 9:**
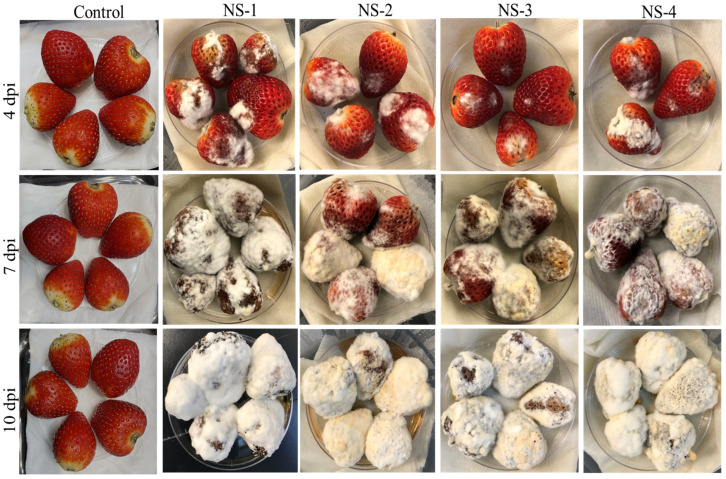
Strawberry fruits infected with four isolates of *Neopestalotiopsis* spp. (NS-1, NS-2, NS-3, and NS-4) at 4, 7, and 10 days post inoculation (dpi). Strawberries in the control group were treated with sterile water only. Sunken lesions containing cottony white mycelium can be observed at 4 dpi, and black acervulli can be observed on strawberry fruits after 7 to 10 dpi.

All *Neopestalotiopsis* spp. isolates produced typical disease symptoms associated with *Pestalotopsis* spp. (Figure 9) on strawberry fruits at three different time points: 4 dpi, 7 dpi, and 10 dpi. Sunken lesions were observed on strawberry fruit after 4 dpi, followed by cottony white mycelial growth on which black acervuli were formed after 7 to 10 dpi. However, the differences among *Neopestalotiopsis* spp. isolates were clearer at 4 dpi than at later time points.

**Figure 10:**
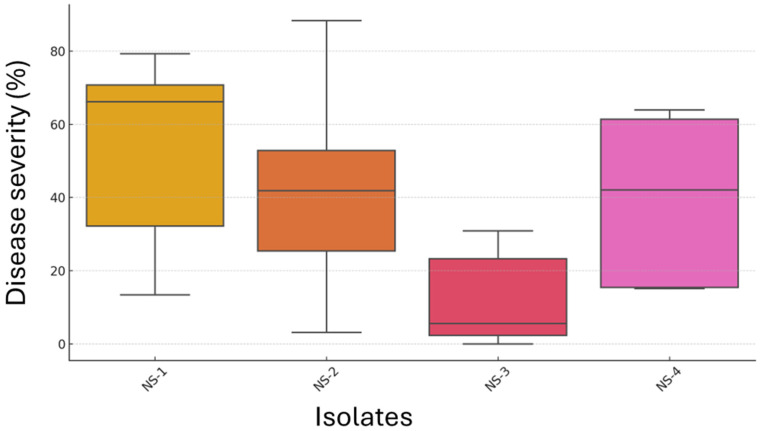
Boxplot showing average disease severity percentage of different *Neopestalotiopsis* spp. isolates (NS-1 to NS-4). Each box represents the interquartile range (IQR), the horizontal line indicates the median, and whiskers represent 1.5× IQR.

Disease incidence for the isolates NS-1 and NS-2 was 100%, whereas for NS-3 and NS-4, it was 80% and 90%, respectively. Furthermore, *Neopestalotiopsis* spp. isolates differed in terms of disease severity percentage (F = 14.12, *p* < 0.0001), where the isolate NS-1 showed the highest average disease severity percentage of 50% (MS), followed by 42.6% (MS) for NS-2, and 39.6% (MS) for NS-4 (Figure 10). However, the isolate NS-3 had an average disease severity percentage of 11.9% (MR), and it differed statistically from the other three isolates (*p* < 0.0001) based on Tukey’s HSD test. No statistical differences were observed for the isolates NS-1, NS-2, and NS-4.

## 4. Discussion

This study aimed to assess the morphological, molecular, and pathogenic diversity of *Neopestalotiopsis* spp. in Nova Scotia, Canada. Morphological and molecular characterization confirmed their close genetic relationship to *Neopestalotiopsis* spp. isolates previously described in Florida and Georgia, particularly isolate 17–43 L from strawberry and several isolates from blueberry [11,24]. We observed variations among *Neopestalotiopsis* spp. isolates when tested on different strawberry cultivars in detached-leaf, potted-plant, and fruit-infection assays. Furthermore, *Neopestalotiopsis* spp. were re-isolated from symptomatic leaves and fruits, fulfilling Koch’s Postulates. This is the first scientific report of the existence of *Neopestalotiopsis* spp. in Nova Scotia, Canada.

The detached-leaf assay revealed clear variation in aggressiveness among the *Neopestalotiopsis* isolates, with NS-1 consistently inducing the most extensive necrosis on both cultivars, with average leaf necrosis of 87.7% on ‘Seascape’ and 88.1% on ‘Albion’ (Figure 5). Likewise, *Neopestalotiopsis* isolate NS-4 was the second most aggressive isolate on both strawberry cultivars, with a moderately susceptible phenotype. In contrast, *Neopestalotiopsis* isolate NS-2 was less aggressive on ‘Seascape’, with average leaf necrosis of 45.5% (MS), but moderately resistant on ‘Albion’, with average leaf necrosis of 16.5% (MR), indicating a potential isolate–cultivar interaction. This subtle variation may reflect quantitative resistance traits that reduce disease severity without conferring complete immunity, as discussed by Baggio et al. [11] in studies of fruit rot resistance. These results mirror observations by Baggio et al. [11], who reported rapid leaf blighting in Florida strawberries, which was associated with *Neopestalotiopsis* spp. isolates phylogenetically related to *N. rosae*. Similar trends were reported by Schierling et al. [26] and Chamorro et al. [6], where *N. rosae* and *N. clavispora* caused progressive leaf necrosis and crown rot. Additionally, Avilés et al. [27] in Spain and Essa et al. [7] in Egypt documented variability in *Neopestalotiopsis* spp. isolates’ aggressiveness across cultivar and tissue type.

In the whole-plant assays, disease severity patterns were broadly consistent with the detached-leaf test, reaffirming the aggressiveness of NS-1 and the relatively reduced aggressiveness of NS-3. All NS isolates tested were moderately aggressive on ‘Honeoye’, similarly to the phenotype observed for all NS isolates on ‘Seascape’ in the detached-leaf assays. In contrast, ‘Albion’ showed a moderately resistant response to NS-2 and NS-3 in both assays, suggesting the presence of inherent genetic resistance to *Neopestalotiopsis* spp. in this cultivar. These observations are consistent with earlier findings by Alam et al. [23] who screened 1578 strawberry advanced breeding lines and reported that 1155 (88%) out of 1316 advanced breeding lines were susceptible to *Neopestalotiopsis* spp. [23]. In the same study, various strawberry varieties, namely Beauty (MS), Brilliance (S), Elyana (MS), Festival (MS), Florida127 (MS), Radiance (S), Medallion (S), and Winterstar (MS), were either moderately susceptible (MS) or susceptible (S) to three *Neopestalotiopsis* spp. isolates [23]. Furthermore, six strawberry varieties, namely Beauty, Festival, Brilliance, Sensation, Camino Real, and Radiance, were reported to be moderately susceptible to highly susceptible to *Neopestalotiopsis* spp. [9,11,28,29,30,31]. In contrast, ‘Honeoye’ was reported to be resistant to *Neopestalotiopsis* spp. isolates from Indiana, further deciphering the existing variability in host response to this newly evolving and emerging species [29].

The fruit-inoculation assays further corroborated isolate-level differences in pathogenicity. The significantly lower disease severity caused by NS-3 (11.9%) compared with *Neopestalotiopsis* isolate NS-1 (50%) at 4 dpi aligns with the fruit-specific pathogenicity dynamics reported by Baggio et al. [11] and Beg et al. [24]. Notably, NS-3 showed low aggressiveness across all assays, suggesting that within the *Neopestalotiopsis* spp., pathotype variation exists, a phenomenon previously documented in polyphyletic genera such as *Colletotrichum* and *Botrytis* [32].

Another key insight from this study is the lack of a complete correlation between phylogenetic clustering and aggressiveness. Although all five Nova Scotia isolates clustered closely with strawberry isolate from Florida (*Neopestalotiopsis* sp. isolate 17–43 L), and the three isolates from Georgia that were originally isolated from blueberry but also pathogenic on strawberry [24], their pathogenicity profiles differed markedly. This observation is consistent with the findings of Maharachchikumbura et al. [2] and Baggio et al. [11], who emphasized that genetic identity alone does not necessarily predict virulence, especially in fungi with high recombination potential or epigenetic plasticity.

Molecular markers such as *ITS*, *TEF1-α*, and *ß-tubulin* were suitable for species delimitation, as previously demonstrated by Maharachchikumbura et al. [2] and Baggio et al. [11]. Nevertheless, the five *Neopestalotiopsis* spp. isolates (NS-1–NS-5) did not cluster with any described *Neopestalotiopsis* species based on available type material such as *N. rosae*, *N. clavispora*, *N. mesopotamica,* and *N. javaensis*, as reported by Baggio et al. [11]. Interestingly, among nine different *Pestalotiopsis* isolates used in phylogenetic tree construction, only *Pestalotiopsis* sp. NA-2014b strain P813 clustered with *Neopestalotiopsis* sp. isolate 16–337. Both *Pestalotiopsis clavispora* isolates clustered together, whereas *Pestalotiopsis foedans* and *P. asiatica* did not cluster with any other species. In addition, *P. rhododendri* strain OP086 clustered with *P. trachycarpicola* strain OP068 (Figure 3).

From an epidemiological perspective, the consistent aggressiveness of NS-1 across detached leaves, potted plants, and strawberry fruits suggests existence of highly virulent *Neopestalotiopsis* spp. isolates capable of initiating infections across multiple host plant tissues under disease-conducive conditions. Such isolates may play a central role in disease establishment and progression within strawberry production systems. This pattern aligns with reports from Florida, where early-season leaf lesions progressed to crown rot and fruit blight [9,11].

The recent recovery of *Neopestalotiopsis* spp. isolates from blueberries in Georgia [24], exhibiting high pathogenic potential on both blueberries and strawberries, represents an alarming situation for strawberry nurseries, fruit producers and blueberry producers in Canada, in particular for Nova Scotia. This risk is amplified by widespread cultivation of *Neopestalotiopsis* spp.-susceptible strawberry varieties across Nova Scotia and other regions of Canada. Nova Scotia’s strawberry industry is quite vibrant, with a farm gate value of about CAD 13.2 million in addition to a strawberry nursery value of about CAD 9 million [1]. Considering the wet spring and humid summer climate of Nova Scotia, the introduction of highly aggressive isolates could pose a serious threat, particularly in fields receiving infected transplants. Our findings indicate a shared phylogenetic lineage between Canadian isolates and those from southeastern U.S., suggesting a possible link to the movement of nursery stock. However, population-level genomic analysis will be required to rigorously test this hypothesis.

Developing resistant cultivars is essential to ensure the long-term sustainability of strawberry production in North America and other regions where *Neopestalotiopsis* spp. pose a recurring and significant threat. A strong source of resistance has been identified in a distant strawberry cultivar, ‘Yasmin’ [23,33]. Interestingly, Alam et al. [23] also conducted genome-wide association analysis using approximately 45,000 SNPs (single-nucleotide polymorphisms) and identified two potential loci, *RNp1* and *RNp2*, associated with moderate resistance to *Neopestalotiopsis* spp. Furthermore, the tightly linked SNP markers identified in that study represent a valuable asset for the accelerated resistance breeding and introgression of the resistant loci into commercially important strawberry varieties [23].

This study highlights the need for further investigations into *Neopestalotiopsis* spp. population dynamics and the disease resistance potential of strawberry cultivars when challenged with isolates with broad virulence spectra. Reliance on a single isolate may lead to underestimation of disease risk, especially when highly virulent *Neopestalotiopsis* isolates such as NS-1 are present in the field. Moreover, the variable host responses across tissue types emphasizes the need for integrated phenotyping strategies, including detached-leaf, potted-plant, and fruit assays, to capture the full spectrum of disease expression.

## 5. Conclusions

This study provides new insights into the pathogenic variability of *Neopestalotiopsis* spp. affecting strawberry production in eastern Canada. The detection and identification of highly aggressive isolates with close genetic similarity to southeastern U.S. strains raises concerns regarding potential transborder movement via infected or asymptomatic plant material. However, additional evidence such as documented plant movement across borders, large-scale regional sampling, and population genome analysis will be required. Continued monitoring, molecular surveillance, and systematic screening of cultivar resistance will be critical steps to mitigate the emerging threat posed by this genetically diverse and adaptable pathogen complex.

## Figures and Tables

**Table 1:** Analysis of variance (ANOVA) showing the effects of cultivar, fungal isolate, and their interaction on necrotic leaf area percentage in strawberry.

	Sum of Squares	df	F Value	PR (>F)	*p*-Value
Cultivar	2298.11	1	23.58	5.17 × 10^−6^	*p* < 0.00001 ***
Isolate	53,213.66	3	181.97	1.30 × 10^−37^	*p* < 0.000000001 ***
Cultivar × Isolate	4407.98	3	15.07	5.35 × 10^−8^	*p* < 0.0000001 ***
Residual	8577.98	88			

Note: Cultivar = strawberry cultivars (Albion, Seascape). Isolate = *Neopestalotiopsis* spp. isolates (NS-1 to NS-4). Cultivar × Isolate = interaction. df = degrees of freedom. PR (>F) = probability associated with the F-statistic; values < 0.05 indicate statistically significant differences. *** *p* < 0.001 (highly significant).

**Table 2:** Analysis of variance (ANOVA) showing differences in disease severity among four *Neopestalotiopsis* spp. isolates tested on strawberry plants of cultivar “Honeoye.”.

	Sum of Squares	df	F Value	PR (>F)	*p*-Value
C (Isolate)	4225	3	7.92	5.20 × 10^−5^	*p* < 0.00001 ***
Residual	34,837	196			

Note: Cultivar = strawberry cultivars (Honeoye). Isolate = *Neopestalotiopsis* spp. isolates (NS-1 to NS-4). df = degrees of freedom. PR (>F) = probability associated with the F-statistic; values < 0.05 indicate statistically significant differences. *** *p* < 0.001 (highly significant).

## Data Availability

The original contributions presented in this study are included in the article/Supplementary Material. Further inquiries can be directed to the corresponding author.

## Supplementary Materials

The following supporting information can be downloaded at: https://www.mdpi.com/article/10.3390/jof12040275/s1, Figure S1. *TEF1-α* sequence alignment. Alignment of partial *TEF1-α* sequences from five *Neopestalotiopsis* spp. (NS-1 to NS-5), three blueberry isolates from Georgia (CB22-023, CB22-027, CB22-028) and *N. rosae* CBS-101057 reveals clear point mutations similar to those in NS-2, CB22-023 (blueberry isolate) and *N. rosae* CBS-101057. Figure S2: In silico analysis of *β-tub* gene sequences of *Neopestalotiopsis* spp. isolates (NS1-NS5) from Nova Scotia were identical with virulent straw-berry isolate from Florida (17–43L), three blueberry isolates from Georgia (CB22-023, CB22-027, CB22-028) but are different from *N. rosae* CBS-101057. Point mutation C to T at 294 bp has been shown with an arrow in the picture. Table S1. GenBank accession numbers of sequences used in the phylogenetic tree in this study. Reference [34] is cited in the supplementary materials.
