# Supplementary material for: Diversity and Pathogenicity of Neopestalotiopsis Species Associated with Strawberry Leaf Spot and Fruit Rot in Nova Scotia
Source: J Fungi (Basel). 2026 Apr 10;12(4):275. doi: 10.3390/jof12040275 (PMC13117497; doi:10.3390/jof12040275)
Supplement: Supplementary file 1 [file jof-12-00275-s001.zip › jof-4127642-supplementary.pdf]

Species	Culture accession/Isolate	Genbank *ITS* Sequence	Genbank *TEF1-α* Sequence	Genbank *β-tub* Sequence	Reference
Pseudopestalotiopsis cocos	CBS 272.29	KM199378	KM199553	KM199467	[2]
Neopestalotiopsis rosae	*97-49F*	MK895141	MK903333	MK903337	[11]
Pestalotiopsis longisetula	173213	AF409971	AB453851	AB453861	Reference Not Available
Neopestalotiopsis clavispora	*TOR-802-803-804*	JQ005776	JQ948677	JQ005860	[6]
Neopestalotiopsis javaensis	*CBS 257.31*	KM199357	KM199543	KM199437	[2]
Neopestalotiopsis rosae	*CBS 124745*	KM199360	KM199524	KM199430	[2]
Neopestalotiopsis rosae strain	CRM-FRH	MN385719	MN268533	MN268530	[34]
Neopestalotiopsis rosae strain	CRM-FRC	MN385718	MN268532	MN268529	[34]
Neopestalotiopsis rosae	*CBS 101057*	KM199359	KM199523	KM199429	[2]
Neopestalotiopsis rosae	*14-691R*	MK895142	MK903334	MK903338	[11]
Neopestalotiopsis	*7927*	KY271740	KY271093	KY271094	Reference Not Available
Neopestalotiopsis rosae	*PEST3*	KY688075	KY688074	KY688073	[7]
Neopestalotiopsis mesopotamica	*CBS 299.74*	KM199361	KM199541	KM199435	[2]
Neopestalotiopsis sp. culture	*NTCC:1324*	PQ573780	PQ594827	PQ594829	Reference Not Available
Neopestalotiopsis sp.	*NTCC:1323*	PQ573249	PQ594826	PQ594828	Reference Not Available
Neopestalotiopsis rosae	*16-337C*	MK895143	MK903335	MK903339	[11]
Pestalotiopsis sp.	*NA-2014b strain P816*	KM074049	KM074050	KM074059	Reference Not Available
Neopestalotiopsis foedans	*CGMCC 3.9123*	JX398987	JX399053	JX399022	[2]
Pestalotiopsis clavispora	*MFLUCC12-0281*	*JX398979*	JX399045	JX399014	[14]
Pestalotiopsis clavispora	*MFLUCC12-0280*	*JX398978*	JX399044	JX399013	[14]
Neopestalotiopsis sp.	*CB22-023*	OR800175	PV492433	PV492420	[24]
Neopestalotiopsis sp.	*17-43L*	MK895144	MK903336	MK903340	[11]
Neopestalotiopsis sp.	*NS2*	ON454614	ON631214	ON549866	This Study
Neopestalotiopsis sp.	*NS1*	ON454613	ON631213	ON464179	This Study
Neopestalotiopsis sp.	*NS3*	ON454615	ON631215	ON549867	This Study
Neopestalotiopsis sp.	*CB22-027*	OR800179	PV492434	PV492421	Reference Not Available
Neopestalotiopsis sp.	*CB22-028*	OR800180	PV492435	PV492422	Reference Not Available
Neopestalotiopsis sp.	*NS4*	ON454616	ON631216	ON549868	This Study
Neopestalotiopsis sp.	*NS5*	ON454617	ON631217	ON549869	This Study
Neopestalotiopsis honoluluana	*CBS 111535*	KM199363	KM199546	KM199461	[2]
Pestalotiopsis asiatica	*MFLUCC12-0286*	JX398983	JX399049	JX399018	[14]
Pestalotiopsis iraniensis	P815 (CBS 137768)	KM074048	KM074051	KM074057	[12]
Pestalotiopsis iraniensis	P814 (CBS 137767)	KM074045	KM074053	KM074056	[12]
Pseudopestalotiopsis indica	*CBS 459.78*	KM199381	KM199560	KM199470	[2]
Pestalotiopsis rhododendri	*OP086*	KC537804	KC537811	KC537818	[2]
Pestalotiopsis trachicarpicola	*Op068*	JQ845947	JQ845946	JQ845945	[2]

Table S1. GenBank accession numbers of sequences used in the phylogenetic tree in this study.

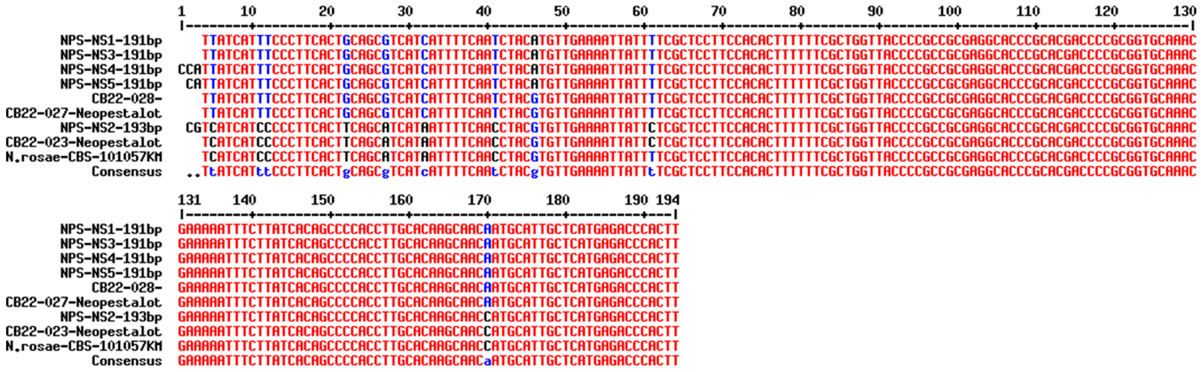

Figure S1. *TEF1-α* sequence Alignment. Alignment of partial *TEF1-α* sequences from five *Neopestalotiopsis* spp. (NS-1 to NS-5), three blueberry isolates from Georgia (CB22-023, CB22-027, CB22-028) and *N. rosae* CBS-101057 reveals clear point mutations similar to those in NS-2, CB22-023 (blueberry isolate) and *N. rosae* CBS-101057.

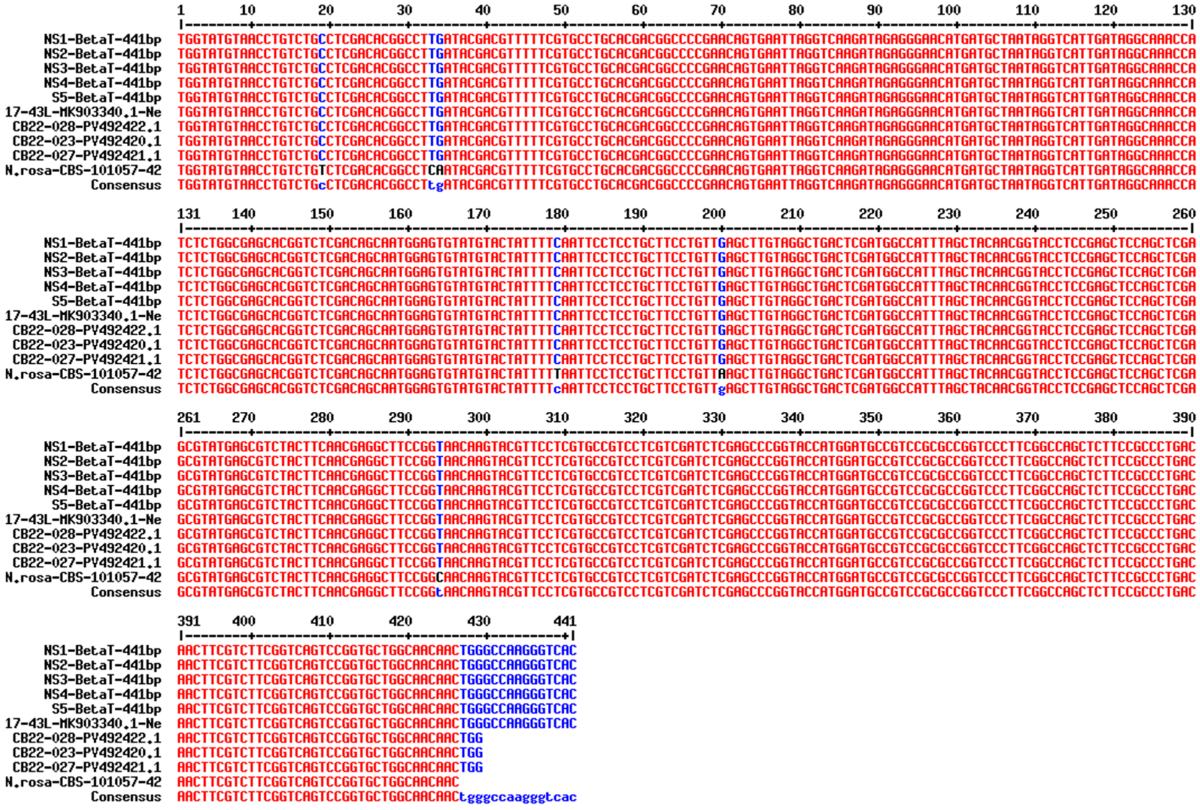

Figure S2: In silico analysis of β-tubulin gene sequences from the Nova Scotia isolates (NS1-NS5) were identical with virulent strawberry isolate from Florida (17-43L), three blueberry isolates from Georgia (CB22-023, CB22-027, CB22-028) but is different from *N. rosae* CBS-101057. Point mutation C to T at 294 bp has been shown with an arrow in the picture.
